# An unexpected intracerebral lesion – case report of a superinfected aspergillosis mimicking a brain metastasis

**DOI:** 10.1186/s12879-021-06176-7

**Published:** 2021-06-07

**Authors:** Basil Erwin Grüter, Anna Maria Reuss, Elisabeth Jane Rushing, Athina Pangalu, Markus Florian Oertel

**Affiliations:** 1 Department of Neurosurgery, University Hospital Zurich, University of Zurich, Frauenklinikstrasse 10, 8091 Zurich, Switzerland; 2 Clinical Neuroscience Center, University Hospital Zurich, University of Zurich, Zurich, Switzerland; 3 Department of Neuropathology, University Hospital Zurich, University of Zurich, Zurich, Switzerland; 4 Department of Neuroradiology, University Hospital Zurich, University of Zurich, Zurich, Switzerland

**Keywords:** Aspergillosis, Bacterial infections and mycoses, Brain abscess, Metastasis, Neuroaspergillosis

## Abstract

**Background:**

Invasive aspergillosis of the central nervous system is a rare but increasingly prevalent disease. We present the unusual case of an immunosuppressed patient suffering from unexpected superinfected invasive aspergillosis with cerebral, pulmonal, and adrenal manifestations, mimicking a metastasized bronchial carcinoma. This report reveals the importance of including aspergillosis in the differential diagnosis of a cerebral mass lesion in the light of unspecific clinical findings.

**Case presentation:**

A 58-year-old immunocompromised female presented to our emergency department with a single tonic-clonic seizure. Imaging showed a ring enhancing cerebral mass with perifocal edema and evidence of two smaller additional hemorrhagic cerebral lesions. In the setting of a mass lesion in the lung, and additional nodular lesions in the left adrenal gland the diagnosis of a metastasized bronchus carcinoma was suspected and the cerebral mass resected. However, histology did not reveal any evidence for a neoplastic lesion but septate hyphae consistent with aspergillus instead and microbiological cultures confirmed concomitant staphylococcal infection.

**Conclusions:**

A high index of suspicion for aspergillus infection should be maintained in the setting of immunosuppression. Clinical and radiological findings are often unspecific and even misleading. Definite confirmation usually relies on tissue diagnosis with histochemical stains. Surgical resection is crucial for establishing the diagnosis and guiding therapy with targeted antifungal medications.

## Background

Invasive aspergillosis of the central nervous system is a rare disease, particularly in the Western World. However, in recent years this entity has become more frequently diagnosed, particularly in immunocompromised patients [1, 2]. Therefore, it is important to include aspergillosis in the potential differential diagnosis of cerebral mass lesion, even in the light of unspecific clinical findings. In this article, we present the unusual case of an immunosuppressed patient suffering from unexpected superinfected invasive aspergillosis with cerebral, pulmonal, and adrenal manifestations, mimicking a metastasized bronchial carcinoma.

## Case presentation

A 58-year-old female presented to our emergency department with new-onset, generalized tonic-clonic seizures controlled by the administration of midazolam and levetiracetam. Her past medical history included a kidney transplantation due to a focal-segmental glomerulosclerosis six months prior to admission, which was treated with immunosuppressive therapy (mycophenolate, prednisone and tacrolimus). In addition, she received levothyroxine substitution after a total thyroidectomy for papillary thyroid cancer 3.5 years ago and she was treated with amlodipine and metoprolol for mild to moderate mitral insufficiency.

**Fig. 1:**
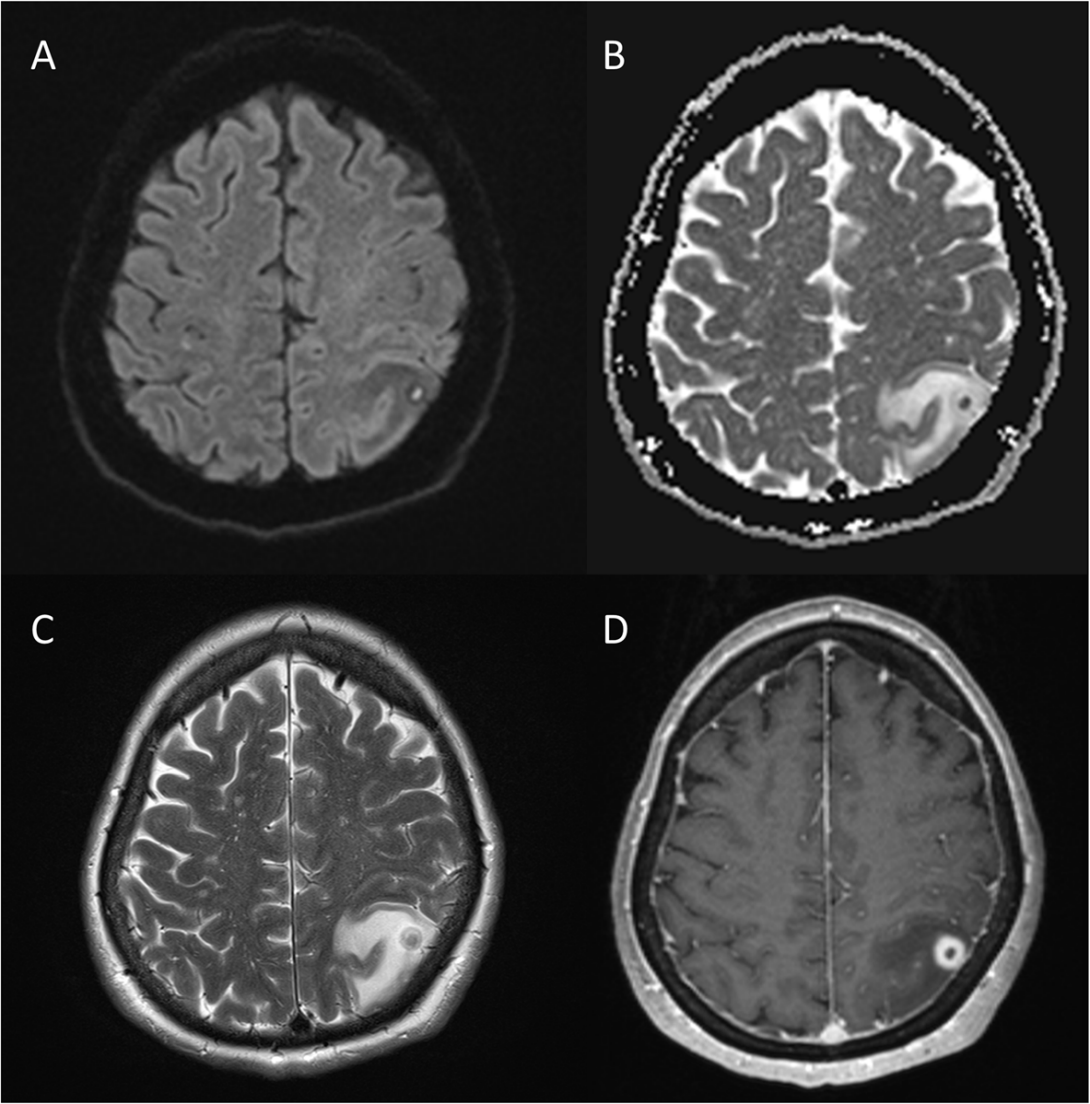
Pre-operative axial MRI findings. Diffusion-weighted images (**a**), with corresponding ADC-map (**b**), showed a diffusion restricted left parietal lesion. On T2-weighted images (**c**) the lesion appeared T2-hyperintense, with both a T2-hypointense central region and thin rim. The lesion showed intense ring-enhancement in the T1-weighted contrast-enhanced images (**d**) and was surrounded by marked perifocal edema (**c**)

On admission, her vital signs were within normal range. An initial aphasia resolved completely within a few hours, and she did not show any focal deficits on neurological examination. A computed tomography (CT) scan of her head revealed a space-occupying lesion in the left parietal lobe. Subsequent magnetic resonance imaging (MRI) tomography of the brain confirmed a ring enhancing mass with central diffusion restriction and perifocal edema in the inferior parietal lobule (Fig. 1). In addition, two smaller, hemorrhagic lesions were visible in the postcentral gyrus and in the centrum semiovale on the right side. In the absence of any clinical evidence of inflammation and unremarkable cerebrospinal fluid (CSF) analysis, brain metastasis was considered the most likely differential diagnosis. Accordingly, staging CT (thorax-abdomen) showed a mass lesion in the right upper lobe of the lung, suspicious of bronchial carcinoma. Additional nodular lesions were seen in the left adrenal gland. Clinically, the patient remained stable under antiepileptic therapy. However, follow-up MRI, four days after the initial examination, showed progression of the left hemispheric lesion and the perifocal edema.

**Fig. 2:**
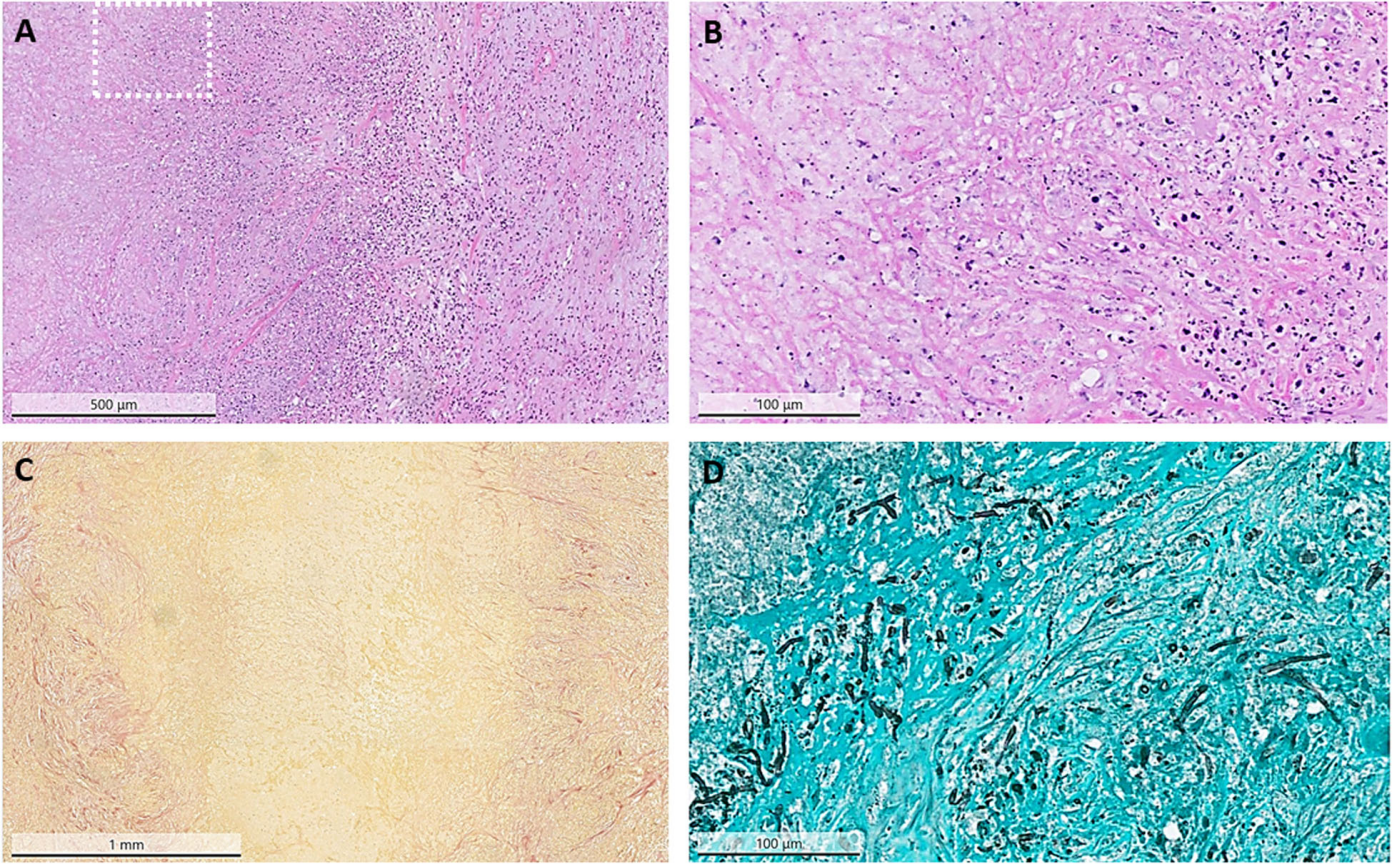
Pathological staining of resected tumor. Histology reveals an abscess containing fungal hyphae surrounded by a collagenous wall. **a** H&E stain shows the abscess wall containing mixed inflammatory cells, including abundant neutrophils. **b** Higher magnification is indicated by the white square in **a**. Hyphae are visible within the abscess wall **c** Elastica-van-Gieson stain demonstrates the collagenous abscess capsule. **d** Grocott silver stain illustrates septate hyphae branching at right angles, consistent with aspergillus

Interdisciplinary consensus was urgent resection of the symptomatic brain lesion, which would also enable a definitive diagnosis. A left parietal craniotomy was performed, and the superficial mass was completely resected. Macroscopically, the lesion appeared firm and indurated without any evidence of pus or necrosis. However, histology did not reveal any evidence for a neoplastic lesion. Instead, a collagenous abscess wall containing mixed inflammatory cells, including abundant neutrophils. The Grocott stain confirmed the presence of branched, septate hyphae consistent with aspergillus (Fig. 2). Panfungal polymerase chain reaction (PCR) linked immunosorbent assay of the resected specimen and fungal cultures remained negative. In view of the clear histological diagnosis of aspergillosis, additional galactomannan antigen tests were not performed.

The postoperative course was uneventful. Therapeutically, voriconazole was initiated, and due to subtherapeutic drug values complemented by amphotericin B. However, the patient developed serious side effects (fever, shivering, nausea and vomitus) from amphotericin B whereupon the antifungal therapy was adapted to isavuconazole which was well tolerated. Initially, the patient received postoperative empiric antibiotics (ceftriaxone and metronidazole), which were adapted to flucloxacilline and metronidazole after microbiological cultures confirmed growth of staphylococcus warneri and pasteuri species. A positron emission tomography (PET)-CT scan showed metabolically active lesions in the right upper lung and left adrenal gland. Because of the yet unclear etiology, interdisciplinary board suggested a video-assisted thoracoscopic surgery (VATS) wedge resection of the right upper lung lobe for diagnostic and therapeutic reasons. Pathological analysis of this tissue confirmed the diagnosis of aspergillus fumigatus although microbiological cultures remained negative. Accordingly, a diagnosis of invasive aspergillosis with cerebral, pulmonal and adrenal manifestations was rendered. Over the course, antibiotics were deescalated to clindamycin, antifungal regime was continued with isavuconazole, and the patient discharged.

## Discussion and conclusion

We present the unusual case of an immunosuppressed patient suffering from unexpected superinfected invasive aspergillosis with cerebral, pulmonal, and adrenal manifestations, mimicking a metastasized bronchial carcinoma.

Central nervous system (CNS) infections with aspergillus are rare and potentially fatal [3, 4]. Microscopically, aspergillus species are characterized by dichotomous branching, septated hyphae [1]. The primary acquisition route for acquiring aspergillus is the inhalation of fungal conidia, which are spores that subsequently reach the upper and lower respiratory tract. Therefore, the paranasal sinus and the lungs are the most common sites of primary aspergillus infection [2]. However, 40% of patients develop extrapulmonary manifestation, with 10–20% demonstrating CNS involvement [4]. The most important risk factors for brain infections are neutropenia and corticosteroid use. In addition, immunocompromised, organ transplant recipients, oncological, hematological, and acquired immune deficiency syndrome patients are at higher risk [2, 4]. Neuroaspergillosis may develop due to extension from the paranasal sinuses and mastoid air cells or by direct penetration after cranial trauma and operations. However, hematogenous dissemination from invasive lung infection is more common in immunocompromised patients [3, 5]. In this population, a mortality rate of > 90% has been reported [4]. Clinical symptoms of an aspergillus infection are unspecific and depend on the location of the fungus. Patients may present with fever, headache, lethargy, altered mental status, seizures, dizziness, gait disorders or other focal neurological deficits [6]. Classically, CSF studies show mononuclear pleocytosis, elevated proteins and normal to reduced glucose values [5]. Microbiological diagnosis of CSF is limited to culture because other methods such as PCR have only been validated in serum and bronchoalveolar lavage specimens [3]. Galactomannan, a polysaccharide antigen located in the cell wall of aspergillus can be detected by enzyme-linked immunosorbent assay [7]. However, we did not perform galactomannan testing, since the diagnosis was already established by histology. Brain imaging is often helpful in establishing the diagnosis, although CT and MR findings are non-specific. Ring-enhancing lesions are not uncommon, but contrast enhancement may be completely absent. Dural enhancement may occur with generalized meningoencephalitis. On unenhanced T1-weighted MRI, aspergillus foci usually appear hypo- to isointense and hypointense on T2-weighted images. Areas of T1-high signal intensity may also be seen in case of hemorrhage. Hemorrhage occurs in approximately 25% of lesions [8, 9]. Furthermore, aspergillus hyphae may invade the walls of small and large blood vessels which results in initial thrombosis, leading to infarction or development of mycotic aneurysms. With regard to therapy, there are only few reports of patients surviving neuroaspergillosis with antifungal medications alone [2]. Importantly, surgical resection must always be complemented with antimycotic medication.

The presented patient was receiving immunosuppressive medications, including corticosteroids (after kidney transplantation). Thus, she harbored an increased risk for developing neuroaspergillosis. Her clinical findings were limited to a single tonic-clonic seizure and imaging features were nonspecific. The presented case illustrates that despite the unspecific clinical findings, it is important to include fungal infections in the differential diagnosis, especially in the setting of immunosuppression from whatever cause. Interestingly, the situation was complicated by a concomitant bacterial infection. The intraoperative findings were atypical in this regard and only microbiological cultures revealed streptococcus infection. Although superinfections are recognized in patients who are immunocompromised undergoing surgery, the detection of a bacterial infection may further confound establishing the definitive diagnosis, as in the presented case. Panfungal PCR from CSF (samples taken before starting the antibiotics) were negative. Although PCR from CSF is considered an experimental diagnostic methodology, it may prove helpful in certain cases. In the present case, however, the definitive diagnosis of aspergillus infection could only be established after histological examination of the resected mass, which is considered the gold standard.

In conclusion, invasive aspergillosis of the CNS is a rare but increasingly prevalent disease, especially in immunocompromised patients. The infection may be isolated or combined with other infectious diseases. Accordingly, a high index of suspicion should be maintained in the setting of immunosuppression. Clinical and radiological findings are often unspecific and even misleading. Definite confirmation usually relies on tissue diagnosis with histochemical stains. Surgical resection is crucial for establishing the diagnosis and guiding therapy with targeted antifungal medications.

## Data Availability

The authors confirm that the data supporting the findings of this study are available within the article. Further clinical data are available on request from the corresponding author, [BEG]. The data are not publicly available due to privacy restrictions of the patient.

## Abbreviations

CSF: Cerebrospinal fluid
CNS: Central nervous system
CT: Computed tomography
MRI: Magnetic resonance imaging
PCR: Polymerase chain reaction
PET: Positron emission tomography
VATS: Video-assisted thoracoscopic surgery
